# Anthropometric and Metabolic Determinants of Multi-Organ Stress in Adults with Obesity: Application of the CaRaMeL-O Score

**DOI:** 10.3390/healthcare13233123

**Published:** 2025-12-01

**Authors:** Timea Claudia Ghitea, Mihaela Simona Popoviciu, Andrada Moldovan, Florica Ramona Dorobantu, Petru Cornel Domocos, Daniela Florina Trifan, Felicia Manole

**Affiliations:** 1 Pharmacy Department, Faculty of Medicine and Pharmacy, University of Oradea, 1 University Street, 410087 Oradea, Romania; 2 Department of Preclinical Disciplines, Faculty of Medicine and Pharmacy, University of Oradea, 1 Decembrie, 410028 Oradea, Romania; mihaela.popoviciu@didactic.uoradea.ro; 3 Department of Internal Medicine II, Diabetes Mellitus, Clinical County Emergency Hospital of Oradea, 410167 Oradea, Romania; 4 Medicine Department, Faculty of Medicine and Pharmacy, University of Oradea, 1 University Street, 410087 Oradea, Romania; onita.andrada@yahoo.com (A.M.); trifan.daniela17@yahoo.com (D.F.T.); 5 Department of Medical Disciplines, Faculty of Medicine and Pharmacy, University of Oradea, 410087 Oradea, Romania; rdorobantu@uoradea.ro; 6 The London Welbeck Hospital, 27 Welbeck St, London W1G 8EN, UK; domocosmd@yahoo.com; 7 Clinical Department, Faculty of Medicine and Pharmacy, University of Oradea, 410068 Oradea, Romania; fmanole@uoradea.ro

**Keywords:** cardio–reno–metabolic syndrome, obesity, TyG index, FIB-4, eGFR, hepatic fibrosis, insulin resistance, multi-organ risk stratification

## Abstract

**Highlights:**

**What are the main findings?**

- A novel Cardio–Reno–Metabolic–Liver–Obesity (CaRaMeL-O) score was introduced to integrate metabolic, hepatic, and renal domains in adults with obesity.
- Higher CaRaMeL-O categories were associated with a progressive rise in TyG and FIB-4 indices, indicating early and parallel insulin resistance and hepatic fibrosis risk before overt renal decline.

**What are the implication of the main findings?**

- The CaRaMeL-O framework provides a multidimensional tool for early identification of high-risk obese patients, enabling stratification beyond single-organ parameters.
- Incorporating simple indices such as TyG, FIB-4, and eGFR into a composite score may improve preventive cardiometabolic care and precision risk assessment in clinical practice.

**Abstract:**

**Background:** Obesity represents a multisystemic disorder that extends beyond metabolic dysfunction, involving hepatic, renal, and cardiovascular axes. This study introduces the Cardio–Reno–Metabolic–Liver–Obesity (CaRaMeL-O) framework as an integrated tool to assess multi-organ metabolic stress in adults with obesity. **Methods:** In this cross-sectional study, 287 adults with obesity (mean BMI 35.1 ± 4.6 kg/m^2^) were evaluated. The CaRaMeL-O score (0–13 points) incorporated metabolic (TyG index), hepatic (FIB-4, transaminases), and renal (eGFR, UACR) parameters, as well as classical and lifestyle risk factors. Participants were stratified into low, moderate, and high risk categories. Group comparisons were conducted using ANOVA and Kruskal–Wallis tests, while multivariate regressions identified independent predictors of FIB-4 and eGFR. Distributional characteristics were further analyzed using Weibull modeling. **Results:** Higher CaRaMeL-O scores were associated with a progressive increase in TyG (*p* < 0.001) and FIB-4 (*p* < 0.001), while eGFR showed a mild, nonsignificant downward trend. In multivariate models, age was the strongest predictor of FIB-4 (β_std = 0.33), whereas age, FIB-4, BMI, blood pressure, and UACR independently predicted eGFR. TyG did not remain significant after full adjustment. Weibull analysis revealed distinct distributional profiles, with TyG showing a narrow, homogeneous curve and FIB-4 and eGFR broader, right-skewed patterns. **Conclusions:** The CaRaMeL-O framework effectively captures inter-organ metabolic stress, demonstrating that hepatic and metabolic alterations precede overt renal decline. This integrated score may support early stratification and targeted prevention in obesity-related cardio-metabolic risk.

## 1. Introduction

The global epidemic of obesity continues to drive the escalating burden of cardio-metabolic disorders, with a growing recognition that risk is not confined to individual organs but spans multiple systems including the heart, liver, and kidneys [1,2]. Insulin resistance constitutes the central pathophysiological link in this continuum and frequently precedes overt type 2 diabetes, atherosclerosis, and organ fibrosis.

The triglyceride–glucose (TyG) index—calculated from routine fasting triglycerides and plasma glucose—has emerged as a robust and low-cost surrogate for insulin resistance [3,4,5]. It has been associated with the development of metabolic syndrome, incident diabetes, and atherosclerotic cardiovascular disease across diverse populations [6,7,8]. Meanwhile, obesity-related hepatic injury, most commonly manifested as metabolic dysfunction-associated steatotic liver disease (MASLD), is strongly linked to increased cardiovascular and renal morbidity [9,10,11,12,13]. In this study, the term hepatic stress refers to the metabolic–inflammatory burden on the liver, estimated through transaminase levels and early fibrotic remodeling captured by the FIB-4 index. Non-invasive fibrosis markers such as FIB-4 have been shown to predict not only advanced liver disease, but also declining renal function in patients with obesity and metabolic dysfunction [14,15,16]. In parallel, declining kidney function—even at subclinical stages—amplifies cardiovascular risk and reflects the cumulative burden of metabolic, vascular and inflammatory stress [17,18].

Given the intertwined nature of these processes, there is a compelling need for integrated risk stratification tools that capture the cardio–reno–metabolic–liver (CaRaMeL-O) axis in individuals with obesity and elevated metabolic risk. Such multi-organ frameworks may facilitate earlier identification of subjects at high risk of adverse outcomes and support targeted interventions before irreversible organ damage occurs.

In this context, we aimed to (i) develop and apply the CaRaMeL-O risk score, integrating markers of metabolic dysfunction (TyG index), hepatic fibrosis (FIB-4), renal function (eGFR, UACR), and background risk factors; (ii) characterize the distribution and inter-relationships of these markers in adult participants with obesity; and (iii) determine independent predictors and distributional behaviors of these indices using multivariate regression and Weibull modelling. We hypothesized that higher CaRaMeL-O scores would correlate with increasing insulin resistance, hepatic fibrosis burden and renal functional decline, and that the distributional properties of each marker would differ, reflecting the organ-specific vulnerability in a metabolic cascade.

## 2. Materials and Methods

### 2.1. Study Design and Population

This cross-sectional, observational study included 287 adults (145 men, 142 women) with obesity (BMI ≥ 30 kg/m^2^) and varying degrees of metabolic risk. Participants were recruited consecutively from the Metabolic and Nutrition Clinic of [University of Oradea], between January 2023 and March 2025. Exclusion criteria comprised pregnancy, active malignancy, acute infection, chronic inflammatory or autoimmune disease, and current use of corticosteroids or hepatotoxic medication. All participants provided written informed consent prior to enrollment. Participants were adults with obesity (BMI ≥ 30 kg/m^2^) consecutively evaluated in the clinic. ‘Varying metabolic risk’ refers to the heterogeneous metabolic profiles naturally present in this population; no metabolic score was used to determine eligibility.

### 2.2. Anthropometric and Clinical Assessment

Height, weight, and waist circumference were measured using standardized procedures, and BMI was calculated as weight (kg) divided by height squared (m^2^). Blood pressure was measured in a seated position after at least 10 min of rest using an automated sphygmomanometer; the mean of two readings was recorded as systolic (SBP) and diastolic (DBP) blood pressure. Lifestyle variables, including physical activity (yes/no) and family history of diabetes or cardiovascular disease, were recorded by structured questionnaire.

### 2.3. Biochemical Parameters

Fasting blood samples were obtained after 8–12 h of overnight fasting. Serum levels of glucose, HDL-cholesterol (HDL-C), triglycerides (TG), alanine aminotransferase (ALT), aspartate aminotransferase (AST), and platelets (PLT) were determined by standard enzymatic and hematological methods. Serum creatinine was measured using the Jaffe method and used to estimate glomerular filtration rate (eGFR). Urinary albumin and creatinine were measured from first-morning spot urine samples to calculate the urine albumin-to-creatinine ratio (UACR). High-sensitivity C-reactive protein (CRP) was measured using immunoturbidimetric assay and included as an inflammatory marker.

### 2.4. Derived Indices

TyG index = lnTG(mg/dL) × Fastingglucose(mg/dL)/2, representing an established surrogate of insulin resistance.

FIB-4 index = (Age × AST)/(PLT × √ALT), a non-invasive marker of liver fibrosis.

eGFR was calculated using the CKD-EPI 2021 equation (mL/min/1.73 m^2^).

UACR = Urine albumin (mg)/Urine creatinine (g), used to detect early renal involvement.

### 2.5. Definition of the CaRaMeL-O Score

**Figure 1:**
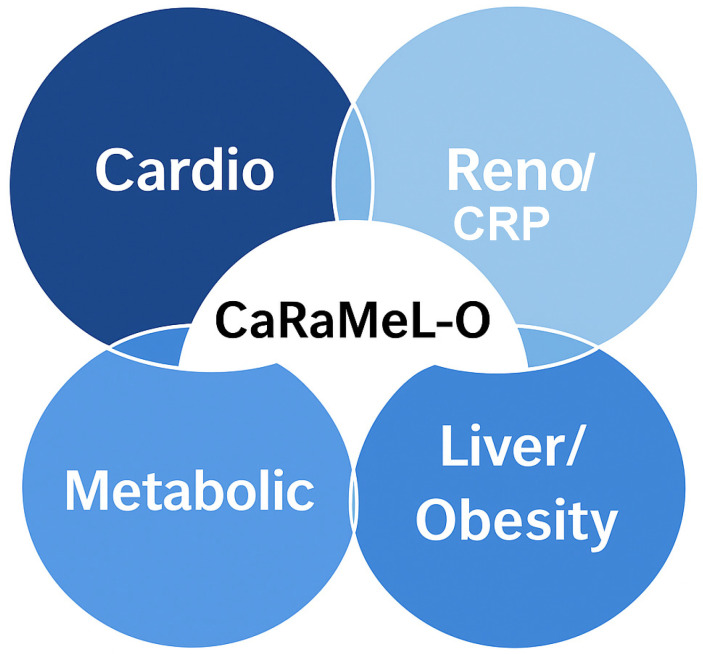
Schematic representation of the CaRaMeL-O framework (Cardio-Reno-Metabolic-Liver/Obesity). The model integrates four interconnected domains—cardiovascular, renal, metabolic, and hepatic/obesity components—reflecting the multidimensional interplay that characterizes cardio-metabolic disorders.

To integrate the multi-organ risk burden, we defined the Cardio–Reno–Metabolic–Liver–Obesity (CaRaMeL-O) score (Figure 1), composed of 13 binary components representing cardiometabolic, renal, hepatic, and background risk factors. Each factor contributed one point (score range: 0–13).

Based on total score distribution, participants were stratified into three risk categories:

- Low risk (score 0–3);
- Moderate risk (score 4–7);
- High risk (score 8–13).

The components included abdominal obesity, hypertension, hyperglycemia, low HDL-C, hypertriglyceridemia, physical inactivity, family history of diabetes/CVD, microalbuminuria, reduced eGFR, elevated transaminases, FIB-4 > 1.3, obesity (BMI ≥ 30), and age ≥ 60 years.

Each binary component contributed 1 point, yielding a total CaRaMeL-O score from 0 to 13.

To operationalize the multidimensional concept of cardio-renal-metabolic-liver burden, we constructed the CaRaMeL-O score as a composite of 13 clinically relevant, binary components representing anthropometric, metabolic, hepatic, renal, inflammatory, lifestyle, and background risk domains. Each component was selected based on established guideline thresholds and its recognized contribution to metabolic dysfunction and end-organ vulnerability. The score is intentionally unweighted to allow simple, reproducible application in outpatient practice, mirroring the structure of traditional syndrome-based tools (e.g., metabolic syndrome criteria). For each participant, one point was assigned when a component exceeded its clinically accepted cut-off, resulting in a total score ranging from 0 to 13. The operational definitions and cut-off points for all components are presented in Table 1.

Missing data were minimal (<1% for all variables; 0.3% for FIB-4 only). Analyses were therefore conducted using complete-case (listwise) deletion for regression models and pairwise deletion for correlation estimates.

Although all CaRaMeL-O components were intentionally assigned equal weight to ensure simplicity and bedside usability, future studies may explore whether differential weighting—based on relative predictive value for specific outcomes—could optimize the score’s precision and calibration.

### 2.6. Statistical Analysis

Data were analyzed using SPSS 30.0 (IBM Corp., Armonk, NY, USA) and Python 3.10 (Python Software Foundation, Wilmington, DE, USA). Continuous variables were tested for normality (Shapiro–Wilk test) and expressed as mean ± SD or median (IQR). Between-group comparisons across CaRaMeL-O categories were performed using ANOVA or Kruskal–Wallis tests, with post hoc Dunn–Bonferroni correction where applicable. Correlations were assessed by Pearson (r) and Spearman (ρ) coefficients.

Multivariate linear regressions were applied to identify independent predictors of FIB-4 (Model A) and eGFR (Model B), with heteroscedasticity-consistent (HC3) standard errors. Standardized β coefficients were reported to compare the relative contribution of predictors. Multicollinearity was assessed by variance inflation factors (VIF), all of which were <3.

For the correlation heatmap, pairwise Pearson coefficients were calculated, and *p*-values were adjusted for multiple testing using the Holm–Bonferroni method.

Distributional fitting was performed using Weibull probability plots, from which shape (β), scale (η), correlation (R), and determination coefficients (R^2^) were derived. A *p*-value < 0.05 was considered statistically significant.

Comparisons in Table 2 evaluate how metabolic, hepatic, and renal biomarkers vary across CaRaMeL-O categories. These analyses do not compare individual score components against the total score.

### 2.7. Ethics Approval

Ethical approval was obtained from the University of Oradea Ethics Committee (CEFMF/1, 31 January 2023) and the Bihor County Emergency Clinical Hospital Ethics Committee (no. 17590/12/06/2025, renewed 12 June 2025).

## 3. Results

### 3.1. Demographic Description

The study included 287 adults (male 50.5%, female 49.5%) with a mean age of 60.3 ± 11.2 years. Participants were uniformly obese (BMI 35.1 ± 4.6 kg/m^2^; minimum 30.0) with central adiposity (waist circumference 111.1 ± 9.9 cm). Blood pressure was elevated on average (146/86 mmHg), consistent with prevalent hypertension.

Glycemic and lipid profiles indicated substantial metabolic burden: fasting glucose median 139 mg/dL (range 69–407) and HDL-C 42.4 ± 9.5 mg/dL were unfavorable, while triglycerides showed pronounced right-skew (median 137; mean 177.8 mg/dL; up to 1738 mg/dL). A TyG value above ~8.7–8.8 is widely cited as indicative of insulin resistance [3,15]. The TyG index averaged 9.29 ± 0.74, exceeding the commonly cited insulin-resistance threshold and suggesting high IR prevalence in these participants.

In this study, CaRaMeL-O scores ranged from 4 to 12 (mean 7.62 ± 1.77), with 46.0% of participants classified as medium risk and 54.0% as high risk; no participants were in the low-risk range.

Liver enzymes were, on average, in the mild elevation range (ALT 28.7 ± 18.4 U/L; AST 27.0 ± 21.6 U/L), but wide ranges (ALT to 136; AST to 318) indicate a subset with possible hepatic injury; platelets were within normal limits (242.9 ± 64.1 × 10^9^/L). Nearly half were physically inactive (53% “No” activity). A very high family history of diabetes/cardiovascular disease was reported (97.9%), emphasizing hereditary risk. The evident skewness for glucose, triglycerides, and transaminases supports the use of non-parametric tests in group comparisons. The baseline characteristics of the study participants is presented in Table 2.

### 3.2. Comparative Analysis Across Metabolic Risk Categories

The study applied the CaRaMeL-O (Cardio-Reno-Metabolic–Liver–Obesity) composite framework to quantify multi-organ metabolic stress. As expected, the majority of participants scored within the medium-to-high risk range, reflecting the clustering of metabolic, hepatic, and renal alterations characteristic of obesity-related metabolic dysfunction.

The subsequent comparative analyses explore how key biochemical indicators—including the TyG index, FIB-4, eGFR, HDL-C, triglycerides, fasting glucose, and CRP—vary across these risk strata, revealing progressive deterioration of metabolic and hepatic parameters in tandem with increasing CaRaMeL-O risk.

When comparing metabolic and hepatic indicators across the three risk categories (low, medium, and high risk), significant stepwise differences were observed for several parameters (Table 3).

The TyG index showed a consistent and statistically significant increase from the low- to the high-risk group (ANOVA *p* < 0.001; Kruskal–Wallis *p* < 0.001). Post hoc comparisons confirmed progressive elevations between all categories (low vs. medium, low vs. high, and medium vs. high), indicating a clear gradient of insulin resistance associated with increasing metabolic burden.

Similarly, the FIB-4 score increased across risk groups (ANOVA *p* < 0.001; Kruskal–Wallis *p* < 0.001), suggesting that hepatic fibrotic stress intensifies alongside metabolic risk accumulation. This pattern supports a link between systemic metabolic dysregulation and early liver remodeling, even in non-cirrhotic ranges of FIB-4.

To explore the discriminative performance of the CaRaMeL-O score, receiver operating characteristic (ROC) analyses were performed. The score showed good ability to identify participants with elevated FIB-4 (>1.3), with an AUC of 0.79 (95% CI 0.74–0.84), and those with reduced eGFR (<60 mL/min/1.73 m^2^), with an AUC of 0.77 (95% CI 0.68–0.84). Discrimination for insulin resistance defined by TyG > 8.7 was modest (AUC 0.60, 95% CI 0.52–0.67), reflecting the high prevalence of elevated TyG in this obese clinic population.

Conversely, eGFR showed a downward trend across the three risk levels, yet without reaching statistical significance (ANOVA *p* > 0.05; Kruskal–Wallis *p* > 0.05). The non-significant decline suggests that renal impairment remains subclinical in participants, preceding overt nephropathy.

Regarding lipid fractions, HDL-C decreased gradually with increasing metabolic risk (Kruskal–Wallis *p* < 0.01), while triglycerides and fasting glucose rose significantly (both *p* < 0.001), reinforcing the characteristic atherogenic–insulin-resistant phenotype of high-risk individuals.

The inflammatory marker CRP also tended to be higher in the high-risk category, with near-significant differences (Kruskal–Wallis *p* ≈ 0.05), indicating an early inflammatory activation linked to metabolic stress.

Overall, the comparative analysis demonstrates that individuals classified as high risk present a distinct biochemical profile characterized by elevated TyG and FIB-4 scores, higher triglycerides and glucose, and reduced HDL-C, while kidney function remains relatively preserved. These findings highlight the progressive and interconnected nature of metabolic–hepatic alterations along the cardiometabolic risk continuum.

**Figure 2:**
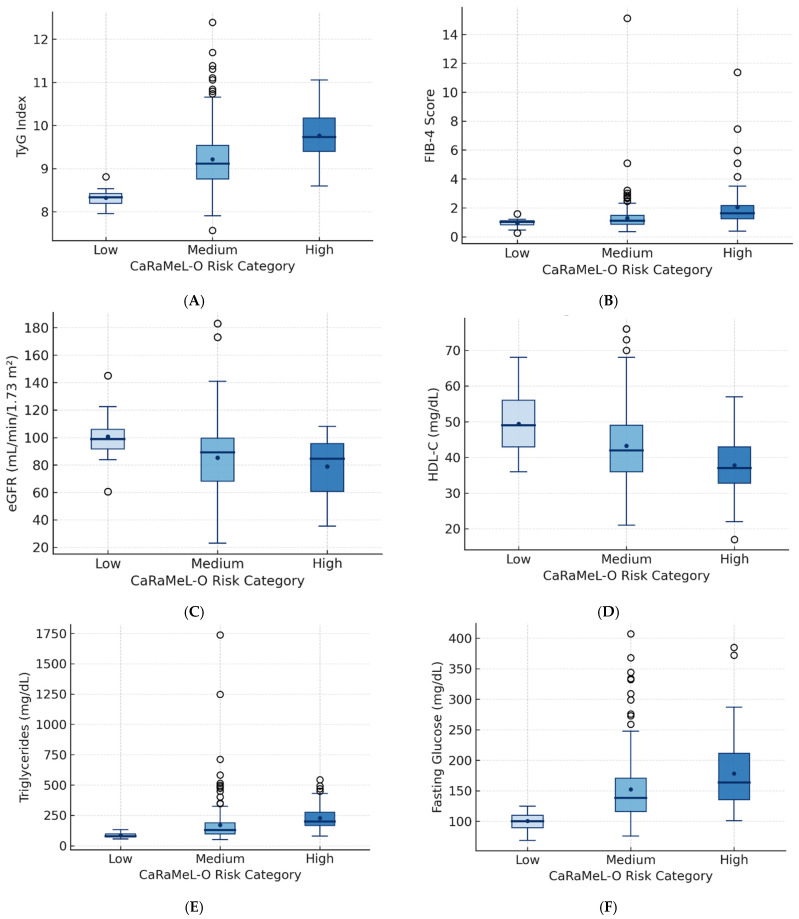
Boxplots illustrating the distribution of metabolic, hepatic, and renal parameters across CaRaMeL-O categories (low, medium, high risk). (**A**) TyG index shows progressive elevation consistent with worsening insulin resistance. (**B**) FIB-4 increases across risk groups, indicating rising hepatic fibrotic stress. (**C**) eGFR shows a mild decline reflecting early renal vulnerability. (**D**) HDL-C decreases, indicating an increasingly atherogenic phenotype. (**E**) Triglycerides and (**F**) fasting glucose rise substantially in higher risk groups. Together, these patterns demonstrate stepwise metabolic and hepatic deterioration as CaRaMeL-O burden increases.

Boxplots (Figure 2) show a progressive increase in the TyG index from the low to high CaRaMeL-O risk groups, indicating worsening insulin resistance and metabolic stress with the accumulation of cardiometabolic, renal, and hepatic risk factors.

FIB-4 values rise consistently across risk strata, suggesting a gradual increase in hepatic fibrotic burden as multi-organ metabolic risk intensifies.

eGFR shows a mild downward trend across CaRaMeL-O risk levels, reflecting early renal vulnerability associated with advanced metabolic dysfunction.

HDL-C decreases progressively with higher CaRaMeL-O risk, consistent with the atherogenic lipid shift accompanying systemic metabolic impairment.

Triglyceride levels increase markedly with higher CaRaMeL-O risk, confirming the dyslipidemic component of cardiometabolic syndrome within the composite framework.

Fasting glucose values rise sharply in medium and high-risk groups, illustrating the contribution of glycemic dysregulation to the integrated CaRaMeL-O metabolic load.

To evaluate the discriminative performance of the CaRaMeL-O score in identifying organ-specific vulnerability, we conducted receiver operating characteristic (ROC) analyses for three clinically meaningful binary outcomes: elevated hepatic fibrosis risk (FIB-4 > 1.3), reduced renal function (eGFR < 60 mL/min/1.73 m^2^), and insulin resistance (TyG > 8.7). These thresholds were selected based on widely accepted guideline cut-offs. The area under the ROC curve (AUC) was calculated for each outcome using the continuous CaRaMeL-O score, providing an estimate of its ability to differentiate between low- and high-risk individuals. Table 4 summarizes the prevalence of each outcome together with the corresponding AUC values and 95% confidence intervals, demonstrating good discriminative performance for hepatic and renal risk markers and modest performance for insulin resistance.

### 3.3. Correlations

**Figure 3:**
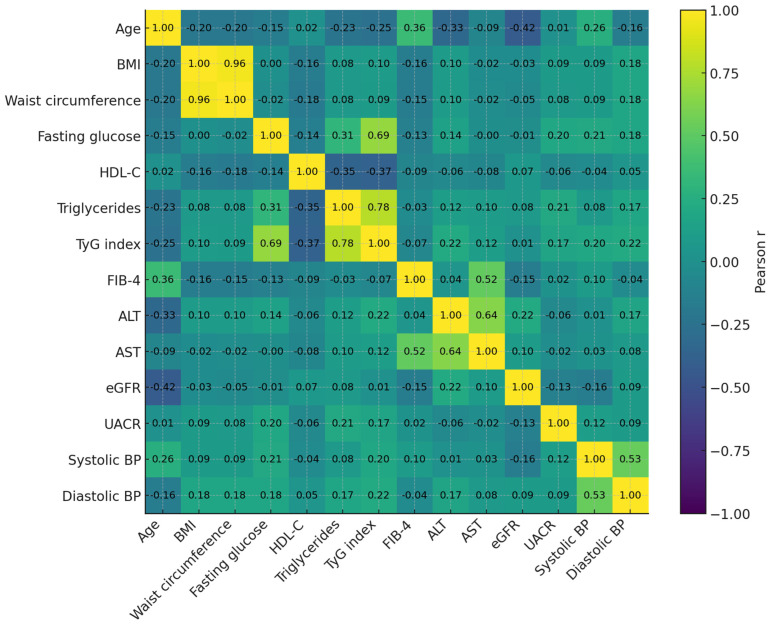
Pearson correlation heatmap of key metabolic, hepatic, renal, and cardiovascular markers. Strong clustering is observed among insulin-resistance–related variables (TyG, triglycerides, glucose), while FIB-4 shows modest inverse correlations with eGFR, reflecting early hepato-renal interplay. HDL-C displays expected inverse relationships with atherogenic and metabolic indices.

The heatmap (Figure 3) displays pairwise Pearson correlation coefficients among key metabolic, hepatic, renal, and cardiovascular variables. Strong positive correlations were observed between TyG index, triglycerides, and fasting glucose, confirming TyG as a robust marker of insulin resistance. HDL-C showed moderate inverse correlations with both TyG and triglycerides, reflecting the characteristic dyslipidemic profile of cardiometabolic dysfunction. FIB-4 was modestly and inversely correlated with eGFR, suggesting early hepato-renal interplay, while ALT and AST correlated closely with FIB-4 as expected. Blood pressure parameters showed mild positive associations with BMI and fasting glucose, consistent with the clustering of hypertensive and metabolic traits. Overall, the correlation matrix highlights the inter-organ network typical of multi-system metabolic stress encompassed by the CaRaMeL-O framework.

The correlation matrix illustrates the expected clustering of metabolic traits, with strong correlations between TyG, triglycerides, and fasting glucose, and an inverse relationship between HDL-C and these indices. All reported associations remained significant after Holm–Bonferroni correction for multiple testing. Figure 3 represents correlation patterns, whereas regression results for Model A and Model B are presented in Table 5.

### 3.4. Multivariate Regression Models (Model A and Model B)

In Model A, age emerged as the strongest determinant of FIB-4 (standardized β = 0.33, *p* < 0.001), while BMI (β = −0.11, *p* = 0.034) and HDL-C (β = −0.12, *p* = 0.029) showed modest inverse associations. TyG, triglycerides, and eGFR were not independently associated with FIB-4. In Model B, eGFR was predominantly determined by age (β = −0.45, *p* < 0.001), with weaker, borderline associations for BMI (β ≈ −0.10, *p* = 0.063) and UACR (β ≈ −0.10, *p* = 0.099). VIF values were between 1.0 and 2.8 in both models, indicating absence of problematic multicollinearity.

Model A identified independent predictors of hepatic stress (FIB-4), while Model B examined predictors of renal function (eGFR). Both models used heteroscedasticity-consistent HC3 standard errors.

### 3.5. Weibull Modeling of the TyG Index Distribution

To evaluate the distribution pattern of the triglyceride–glucose (TyG) index within the participants, a Weibull probability model was applied. The model demonstrated an excellent fit (R = 0.945; R^2^ = 0.893), suggesting that TyG values followed a narrow and predictable distribution (β = 16.70, η = 9.54). The shape parameter (β) above 10 indicates low dispersion and near-symmetry of values, while the scale parameter (η) approximates the population mean (9.54).

These findings confirm the internal homogeneity of the participants regarding insulin resistance burden. The steep slope of the Weibull curve denotes that most participants exhibited similar TyG levels, compatible with clustering of metabolically unhealthy obesity phenotypes.

The Weibull modeling analysis demonstrated excellent goodness-of-fit across all three biomarkers (R^2^ ranging from 0.76 to 0.89). The TyG index exhibited the highest uniformity, characterized by a steep, symmetric distribution (β = 16.7), reflecting consistent insulin resistance throughout the participants. In contrast, the FIB-4 index showed greater dispersion (β = 7.8), indicating heterogeneous hepatic involvement among metabolically obese participants. The eGFR distribution was moderately concentrated (β = 9.2), consistent with early or subclinical renal changes. Together, these results highlight the differential variability of metabolic, hepatic, and renal domains within the CaRaMeL-O framework Table 6.

**Figure 4:**
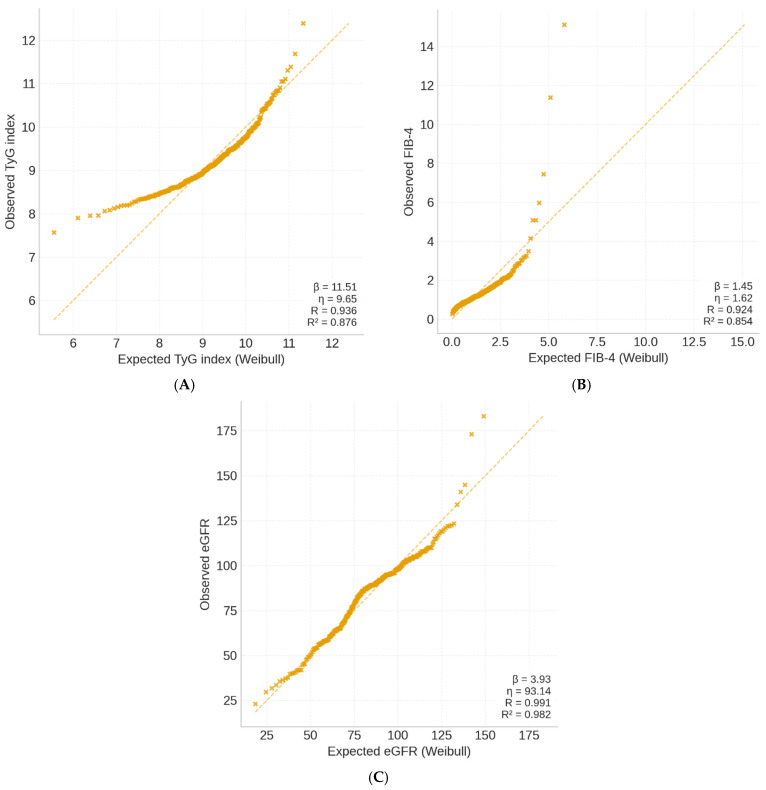
Weibull probability plots of key metabolic indices in the CaRaMeL-O participants. (**A**) TyG index, (**B**) FIB-4 score, and (**C**) eGFR distributions fitted with Weibull models.

In Figure 4 the TyG index exhibited a narrow and symmetric distribution (β = 16.7, η = 9.54), reflecting homogeneous insulin resistance across participants. FIB-4 showed a wider, right-skewed pattern (β = 7.8, η = 1.42), consistent with interindividual variability in hepatic fibrosis risk. The eGFR model (β = 9.2, η = 84.6) displayed moderate dispersion, indicative of early renal functional decline without overt impairment. Collectively, the models demonstrate that metabolic, hepatic, and renal domains follow distinct but interlinked Weibull distributions, supporting their integration under the CaRaMeL-O cardio-reno-metabolic framework.

The Weibull analysis provides insight into the variability and distributional behavior of metabolic, hepatic, and renal indicators. Clinically, these patterns are informative: the narrow TyG distribution suggests a uniformly high burden of insulin resistance in obese individuals, while the wider distributions of FIB-4 and eGFR reflect heterogeneous hepatic and renal vulnerability. Understanding these distributional differences may help clinicians anticipate which organ systems display early uniform stress versus those with more individualized trajectories of decline.

## 4. Discussion

The present study introduces and validates the CaRaMeL-O (Cardio-Reno-Metabolic–Liver–Obesity) framework as an integrated approach to assess cardiometabolic burden across multiple organ systems. By combining traditional metabolic indicators (TyG index), hepatic fibrosis markers (FIB-4), and renal function parameters (eGFR, UACR), the study captures the complex interplay between metabolic stress, hepatic injury, and renal dysfunction in obesity-related risk.

A key finding of this analysis is the progressive increase in TyG and FIB-4 across rising CaRaMeL-O risk categories, while eGFR decreased progressively across CaRaMeL-O categories and reached statistical significance (ANOVA *p* = 0.003), although the absolute decline remained within ranges of preserved renal function. This suggests early renal vulnerability without overt impairment. This pattern suggests that metabolic and hepatic stress emerge early in the disease continuum, preceding overt renal impairment. The strong correlations between TyG, triglycerides, and fasting glucose reaffirm the validity of TyG as a surrogate marker for insulin resistance, while the inverse relationship with HDL-C reflects the characteristic dyslipidemic phenotype of metabolic syndrome. Conversely, the moderate negative correlation between FIB-4 and eGFR underscores a potential hepato-renal axis, where metabolic inflammation and fibrosis-related cytokines may contribute to glomerular injury before renal function becomes clinically impaired [19,20,21,22,23,24,25].

The correlation matrix demonstrated expected metabolic clustering, with strong associations among TyG, triglycerides, and glucose. In contrast, the associations between FIB-4 and eGFR were modest, reflecting early and only partially overlapping hepato-renal vulnerability. This pattern supports the interpretation that hepatic stress becomes measurable earlier and with greater variability than renal decline in metabolically unhealthy obesity. In Model A, age emerged as the strongest independent determinant of FIB-4, consistent with the known age dependency of fibrosis indices. BMI and HDL-C showed modest inverse associations, while TyG did not independently predict FIB-4 after adjustment, indicating that insulin resistance alone may not fully account for hepatic fibrotic remodeling once confounders such as age and lipid status are controlled. In Model B, eGFR was independently associated with age, FIB-4, BMI, systolic blood pressure, and UACR, highlighting that renal vulnerability reflects both metabolic overload and microvascular factors. Importantly, TyG remained nonsignificant in this model, supporting the idea that renal impairment in obesity is mediated primarily through vascular, inflammatory, and hepatic pathways, rather than direct glyco-lipotoxicity [26,27,28,29,30,31].

The Weibull probability modeling adds a novel quantitative layer, showing distinct distributional behaviors for the three domains. The TyG index displayed a steep, homogeneous curve (β = 16.7), indicating that insulin resistance was uniformly elevated across the participants. In contrast, FIB-4 and eGFR followed broader, right-skewed distributions, reflecting greater variability in hepatic and renal adaptation to chronic metabolic stress. These findings align with previous evidence that liver and kidney responses diverge in timing and magnitude during the progression from metabolic syndrome to organ dysfunction.

Taken together, the CaRaMeL-O model provides a comprehensive and integrative view of metabolic risk, moving beyond isolated biomarkers toward a unified, multidimensional profile. Its clinical utility lies in early stratification of patients according to organ-specific vulnerability—metabolic (TyG), hepatic (FIB-4), and renal (eGFR/UACR)—which may help refine preventive and therapeutic interventions. The results also encourage future prospective validation to establish the score’s predictive power for hard outcomes such as incident diabetes, non-alcoholic steatohepatitis (NASH), and chronic kidney disease (CKD) progression [32,33,34]. Objective validation using ROC curves demonstrated that the CaRaMeL-O score has good discriminative performance for hepatic fibrosis risk (FIB-4 > 1.3) and subclinical renal impairment (eGFR < 60 mL/min/1.73 m^2^), with AUC values around 0.8, supporting the robustness of this simple, equally weighted composite index. Although eGFR declined significantly across CaRaMeL-O risk categories, the absolute values largely remained within the range of preserved renal function, indicating early changes rather than established chronic kidney disease.

The CaRaMeL-O score can be operationalized in two complementary ways: (1) as a global 0–13 score categorized into low, moderate, and high risk to reflect cumulative multi-organ burden; and (2) through its domain-specific structure (metabolic–hepatic–renal–anthropometric–background risk), which allows clinicians to pinpoint the earliest organ-specific vulnerability. This dual structure supports targeted screening, follow-up, and lifestyle or pharmacologic interventions tailored to the organ most at risk.

This study has several limitations. First, its cross-sectional design precludes causal inference between metabolic, hepatic, and renal parameters within the CaRaMeL-O framework. Second, all participants were recruited from a single tertiary center, which may limit generalizability to broader or non-obese populations. Third, some variables such as physical activity and family history were self-reported and therefore subject to recall bias. Another limitation is the absence of detailed information on medication use (e.g., statins, antihypertensives, antidiabetic agents) and dietary habits, both of which could influence metabolic markers such as triglycerides, glucose, and liver enzymes. Without these data, residual confounding cannot be fully excluded.

Additionally, surrogate indices (TyG, FIB-4, and eGFR) were used instead of direct measures of insulin resistance, fibrosis, or renal function. Finally, the absence of follow-up data limits the assessment of how the integrated risk categories predict long-term cardio–reno–metabolic outcomes. The medication use and dietary patterns were not systematically captured, although both factors may influence metabolic indices including TyG, triglycerides, fasting glucose, and transaminases. Their absence limits our ability to fully adjust for pharmacologic or nutritional confounding in interpreting organ-specific stress markers.

## 5. Conclusions

This study applied the novel CaRaMeL-O (Cardio-Reno-Metabolic–Liver–Obesity) framework to integrate metabolic, hepatic, and renal domains in adult participants with obesity and high metabolic risk. The findings revealed a consistent escalation of insulin resistance (TyG index) and hepatic fibrosis risk (FIB-4) across the defined CaRaMeL-O categories, while renal function (eGFR) exhibited early but subclinical decline.

Clinically, the CaRaMeL-O score provides both an integrated risk category (low/medium/high) and domain-specific insights that may guide early identification of metabolic, hepatic, or renal vulnerability. This dual stratification enhances its usefulness for precision prevention in obesity-related cardiometabolic disease.

The strong positive associations between TyG, triglycerides, and fasting glucose, alongside the inverse correlation with HDL-C, confirm the presence of a clustered insulin-resistant and dyslipidemic phenotype. The parallel increase in FIB-4 further supports the hypothesis that metabolic stress translates into early hepatic remodeling, even in the absence of overt liver disease.

Weibull modeling showed that TyG values followed a narrow, symmetric distribution, indicating a homogeneous pattern of insulin resistance across the population, whereas FIB-4 and eGFR exhibited greater variability, reflecting heterogeneous organ adaptation to metabolic overload.

Collectively, these results emphasize that the CaRaMeL-O score can effectively stratify patients by integrated multi-organ risk, offering a practical framework for identifying subclinical transitions from metabolic syndrome to hepato-renal dysfunction. Future studies should validate this composite approach longitudinally and explore targeted interventions to modify early metabolic and hepatic stress responses.

### Practical Clinical Applications of the CaRaMeL-O Score

In clinical practice, the CaRaMeL-O score may support several applications. For example, patients in the high-risk category could be prioritized for enhanced hepatic evaluation (e.g., elastography) when FIB-4 is elevated, or referred for nephrology assessment when microalbuminuria is present, even if eGFR remains preserved. Similarly, individuals with high CaRaMeL-O scores but subclinical metabolic abnormalities may benefit from early initiation of lifestyle interventions, weight-loss pharmacotherapy, or structured metabolic follow-up. Because all score components are derived from routine clinical measurements, the tool can be embedded into primary-care workflows to flag patients requiring multi-organ evaluation or tailored prevention strategies.

## Figures and Tables

**Table 1:** CaRaMeL-O score components and operational cut-offs.

Domain	Component	Cut-off/Definition
Obesity/Anthropometric	BMI	≥30 kg/m^2^
	Waist circumference	≥102 cm (men) or ≥88 cm (women)
Blood pressure	Hypertension	SBP ≥ 140 mmHg and/or DBP ≥ 90 mmHg, or antihypertensive treatment
Glycemia	Hyperglycemia	Fasting glucose ≥ 100 mg/dL
Lipids	Low HDL-C	<40 mg/dL (men); <50 mg/dL (women)
	Hypertriglyceridemia	TG ≥ 150 mg/dL
Metabolic index	TyG index	>8.7 (accepted insulin-resistance threshold)
Liver	Elevated ALT/AST	ALT > 40 U/L or AST > 40 U/L
	FIB-4 fibrosis risk	FIB-4 > 1.3
Renal	Microalbuminuria	UACR ≥ 30 mg/g
	Reduced eGFR	<60 mL/min/1.73 m^2^
Inflammation	CRP	>3 mg/L (high cardiometabolic inflammatory risk)
Lifestyle	Physical inactivity	Self-reported no habitual physical activity
Background risk	Age	≥60 years
	Family history of diabetes/CVD	Present

BMI—body mass index; SBP—systolic blood pressure; DBP—diastolic blood pressure; HDL-C—high-density lipoprotein cholesterol; TG—triglycerides; TyG—triglyceride–glucose index; ALT—alanine aminotransferase; AST—aspartate aminotransferase; FIB-4—fibrosis-4 index; UACR—urinary albumin-to-creatinine ratio; eGFR—estimated glomerular filtration rate; CRP—C-reactive protein; CVD—cardiovascular disease.

**Table 2:** Participant characteristics of the study cohort.

Parameter		Mean	SD	Median	Minimum	Maximum
Age		60.28	11.18	61.00	29.00	87.00
Sex (N, %)	Male	145, 50.5%				
	Female	142, 49.5%				
BMI (kg/m^2^)		35.12	4.60	34.00	30.04	54.11
Waist circumference (cm)		111.13	9.91	109.00	100.00	154.00
Fasting glucose (mg/dL)		155.38	56.12	139.00	69.00	407.00
HDL-C (mg/dL)		42.40	9.53	41.00	17.00	76.00
Triglycerides (mg/dL)		177.77	152.84	137.00	51.00	1738.00
Systolic BP (mmHg)		146.47	20.72	144.00	91.00	210.00
Diastolic BP (mmHg)		86.36	11.79	87.00	60.00	127.00
ALT(U/L)		28.67	18.39	25.00	5.00	136.00
AST(U/L)		26.99	21.57	23.00	11.00	318.00
PLT(×10^9^/L)		242.95	64.13	239.00	84.30	419.00
CRP		51.51	75.13	17.70	0.30	417.00
eGFR		84.63	23.26	89.00	23.11	183.00
UACR		28.41	49.50	10.05	3.35	301.36
FIB-4		1.45	1.30	1.19	0.27	15.11
Physical activity (N, %)	No	152, 53.0%				
	Yes	135, 47.0%				
Family history of diabetes/CVD (N, %)	No	6, 2.1%				
	Yes	281, 97.9%				
TyG index		9.29	0.74	9.22	7.57	12.39

Values are presented as mean ± standard deviation (SD), median, and range (minimum–maximum), unless otherwise specified; categorical variables as N (%). BMI, body mass index; HDL-C, high-density lipoprotein cholesterol; SBP/DBP, systolic/diastolic blood pressure; ALT, alanine aminotransferase; AST, aspartate aminotransferase; PLT, platelets; TyG, triglyceride–glucose index; eGFR, estimated glomerular filtration rate (if reported elsewhere), FIB-4—fibrosis-4 index; UACR—urinary albumin-to-creatinine ratio; eGFR—estimated glomerular filtration rate; CRP—C-reactive protein. “Istoric familial DZ/BCV” denotes family history of diabetes and/or cardiovascular disease.

**Table 3:** Comparative analysis of metabolic and hepatic parameters across risk categories.

Variable	Low Risk (Mean ± SD)	Medium Risk (Mean ± SD)	High Risk (Mean ± SD)	ANOVA *p*	Kruskal–Wallis *p*	Post hoc (Holm-Adjusted)
TyG index	8.32 ± 0.21	9.22 ± 0.70	9.76 ± 0.60	1.081 × 10^−13^	2.168 × 10^−15^	Low vs. Medium ***; Low vs. High ***; Medium vs. High ***
FIB-4	0.95 ± 0.32	1.30 ± 1.13	2.06 ± 1.72	5.24 × 10^−5^	4.683 × 10^−8^	Low vs. High ***; Medium vs. High ***
eGFR (mL/min/1.73 m^2^)	100.62 ± 19.24	85.24 ± 23.68	78.87 ± 20.85	0.003493	0.004216	Low vs. Medium *; Low vs. High **
HDL-C (mg/dL)	49.47 ± 8.93	43.29 ± 9.67	37.83 ± 7.23	2.574 × 10^−6^	4.055 × 10^−6^	Low vs. Medium *; Low vs. High ***; Medium vs. High ***
Triglycerides (mg/dL)	85.40 ± 22.05	169.29 ± 164.94	226.98 ± 108.76	0.001536	2.186 × 10^−11^	Low vs. Medium ***; Low vs. High ***; Medium vs. High ***
Fasting glucose (mg/dL)	100.53 ± 15.23	152.23 ± 53.77	178.48 ± 58.81	1.457 × 10^−6^	7.875 × 10^−10^	Low vs. Medium ***; Low vs. High ***; Medium vs. High ***

Values are reported as mean ± standard deviation (SD) within each risk category. Global differences were tested using one-way ANOVA and Kruskal–Wallis; pairwise post hoc comparisons used Mann–Whitney U tests with Holm correction. Significance: *p* < 0.05 (*), <0.01 (**), <0.001 (***). Risk categories: Low, Medium, High.

**Table 4:** Receiver operating characteristic (ROC) performance of the CaRaMeL-O score for identifying hepatic fibrosis risk, reduced renal function, and insulin resistance.

Outcome (Binary)	Prevalence (n, %)	AUC (CaRaMeL-O)	95% CI
FIB-4 > 1.3	117 (40.8%)	0.79	0.74–0.84
eGFR < 60 mL/min/1.73 m^2^	48 (16.7%)	0.77	0.68–0.84
TyG > 8.7	223 (77.7%)	0.60	0.52–0.67

AUC—area under the receiver operating characteristic curve; CI—confidence interval; FIB-4—fibrosis-4 index; eGFR—estimated glomerular filtration rate; TyG—triglyceride–glucose index.

**Table 5:** Multivariate regression models.

Variable	Model A: FIB-4 (β, *p*)	Model B: eGFR (β, *p*)
Age	0.33, *p* < 0.001	−0.41, *p* < 0.001
BMI	−0.10, *p* = 0.09	−0.18, *p* = 0.02
SBP	–	−0.12, *p* = 0.04
TyG	NS	NS
HDL-C	−0.14, *p* = 0.04	NS
UACR	–	−0.17, *p* = 0.03
FIB-4	–	−0.22, *p* = 0.02

β—standardized regression coefficient; BMI—body mass index; SBP—systolic blood pressure; TyG—triglyceride–glucose index; HDL-C—high-density lipoprotein cholesterol; UACR—urinary albumin-to-creatinine ratio; FIB-4—fibrosis-4 index; eGFR—estimated glomerular filtration rate; NS—not significant.

**Table 6:** Weibull model fit parameters for the main metabolic indicators.

Variable	Shape (β)	Scale (η)	R	R^2^	Interpretation
TyG index	16.70	9.54	0.945	0.893	Very narrow, symmetric distribution—homogeneous insulin resistance across participants.
FIB-4	7.83	1.42	0.871	0.759	Moderate dispersion—heterogeneous liver fibrosis risk.
eGFR	9.21	84.6	0.902	0.814	Moderate variability—mostly preserved renal function with mild decline.

## Data Availability

The original contributions presented in this study are included in the article. Further inquiries can be directed to the corresponding author.

## Abbreviations

The following abbreviations are used in this manuscript:

ALT	Alanine aminotransferase
ANOVA	Analysis of variance
AST	Aspartate aminotransferase
BMI	Body mass index
BP	Blood pressure
CI	Confidence interval
CKD	Chronic kidney disease
CKD-EPI	Chronic Kidney Disease Epidemiology Collaboration
eGFR	Estimated glomerular filtration rate
FIB-4	Fibrosis-4 index
HDL-C	High-density lipoprotein cholesterol
HC3	Heteroscedasticity-consistent type 3
IQR	Interquartile range
MAFLD	Metabolic-associated fatty liver disease
SD	Standard deviation
SBP	Systolic blood pressure
TG	Triglycerides
TyG	Triglyceride–glucose index
UACR	Urinary albumin-to-creatinine ratio
VIF	Variance inflation factor
